# DBScope as a versatile computational toolbox for the visualization and analysis of sensing data from deep brain stimulation

**DOI:** 10.1038/s41531-024-00740-z

**Published:** 2024-07-15

**Authors:** Andreia M. Oliveira, Eduardo Carvalho, Beatriz Barros, Carolina Soares, Manuel J. Ferreira-Pinto, Rui Vaz, Paulo Aguiar

**Affiliations:** 1 Neuroengineering and Computational Neuroscience Lab, Instituto de Investigação e Inovação em Saúde (i3S) - University of Porto, Porto, Portugal; 2 Faculty of Engineering of University of Porto (FEUP), Porto, Portugal; 3 ICBAS School of Medicine and Biomedical Sciences - University of Porto, Porto, Portugal; 4 Faculty of Medicine of University of Porto (FMUP), Porto, Portugal; 5 Centro Hospitalar Universitário de São João (CHUSJ), Porto, Portugal

**Keywords:** Parkinson's disease, Predictive medicine

## Abstract

Different neurostimulators for deep brain stimulation (DBS) come already with the ability to chronically sense local field potentials during stimulation. This invaluable new data has the potential to increase our understanding of disease-related brain activity patterns, their temporal evolution, and their modulation in response to therapies. It also gives the opportunity to unveil new electrophysiological biomarkers and ultimately bring adaptive stimulation therapies closer to clinical practice. Unfortunately, there are still very limited options on how to visualize, analyze, and exploit the full potential of the sensing data from these new DBS neurostimulators. To answer this need, we developed a free open-source toolbox, named DBScope, that imports data from neurostimulation devices and can be operated in two ways: via user interface and programmatically, as a library of functions. In this way, it can be used by both clinicians during clinical sessions (for instance, to visually inspect data from the current or previous in-clinic visits), and by researchers in their research pipelines (e.g., for pre-processing, feature extraction and biomarker search). All in all, the DBScope toolbox is set to facilitate the clinical decision-making process and the identification of clinically relevant biomarkers. The toolbox is already being used in clinical and research environments, and it is freely available to download at GitHub (where it is also fully documented).

## Introduction

Deep Brain Stimulation is an established practice for the treatment of neurologic diseases and shows promising prospects for psychiatric conditions^1,2^. It involves the delivery of electrical stimulation to targeted brain regions, that vary in respect to the disease in question^3–5^. While the exact mechanisms of action underlying DBS remain unknown, evidence supports its capacity to modulate neural circuits locally and non-locally^5,6^. Current DBS therapies are delivered in a continuous and open-loop fashion: using a trial and error approach, the clinician selects the best performing stimulation parameters (i.e., a combination of higher symptom mitigation and lower incidence of adverse effects) from established therapeutic parameter ranges^7–9^; after selection, the parameters are kept until the following clinical session, where they are re-adjusted.

Medtronic, a leading company in DBS, announced in 2020 the first US/EU approved neurostimulator with the ability to chronically sense local field potentials during stimulation (Percept PC)^10^. Since then, clinicians and researchers have been integrating the retrieval of the recorded sensing data (using a ‘programming’ tablet) into their practices, although with clinicians doing so in a more gradual manner. This inclusion has been key in increasing our understanding of disease-related brain activity patterns, their temporal evolution, and their modulation in response to therapies. In addition, it also gave the opportunity to unveil new electrophysiological biomarkers and ultimately bring the implementation of adaptive stimulation therapies closer to clinical practice^11–13^. Nonetheless, the current visualization tools accessible on the clinician’s tablet are limited: they fall short in fully exploiting the information embedded within the recordings and are ill-suited for longitudinal analyses (reviewing current and previous data). Consequently, clinicians and researchers have been compelled to seek and develop custom-made software to handle the files exported from the tablet.

Until recently, very few toolboxes were created for this purpose and addressed only simple extraction and visual methods for specific types of recordings^13–15^. Among these resources, the Perceive toolbox stands out for its capability to import Percept PC recordings and make the data available in MATLAB (MathWorks, Natick, MA), allowing the user to interact with the data programmatically^15^. It is also capable of exporting the data in a format compatible with Fieldtrip, an open-source toolbox that is commonly used for MEG, EEG and iEEG analysis^16^. Still, all these toolboxes provide limited intrinsic functionality and require expertise in programming, a deterrent factor for many clinicians/researchers working on DBS. Moreover, the use of tools tailored to other electrophysiological signals fails to fully leverage the specificities of DBS data. These shortcomings have recently been challenged by a new open-source platform called BRAVO (Brain Recording Analysis and Visualization Online), which using a web-based interface offers visual tools for DBS data analysis^17^. While this platform addresses the ‘parsing challenge’ and allows the concurrent analysis of several files, its use poses technical challenges (requires configuration of a Linux server), and special attention regarding clinical data sharing/security (non-local data analysis).

In short, there is still a pressing need for easy-to-use yet versatile tools that facilitate the visualization and analysis of the data downloaded from currently available neurostimulators. To answer this need, we developed a free open-source toolbox, named DBScope, that imports data from neurostimulation devices and can be operated in two ways: via user interface and programmatically, as a library of functions. In this way, it can be used by both clinicians during clinical sessions (for instance, to visually inspect data from the current or previous in-clinic visits), and by researchers in their research pipelines (e.g., for pre-processing, feature extraction and biomarker search). At this stage, DBScope contains a parser for the Percept PC device, but as other devices with sensing capabilities become available, other data parsers can be included. All in all, the DBScope toolbox is set to facilitate the clinical decision-making process and the identification of clinically relevant biomarkers.

## Results

### User-friendly graphical interface

**Fig. 1:**
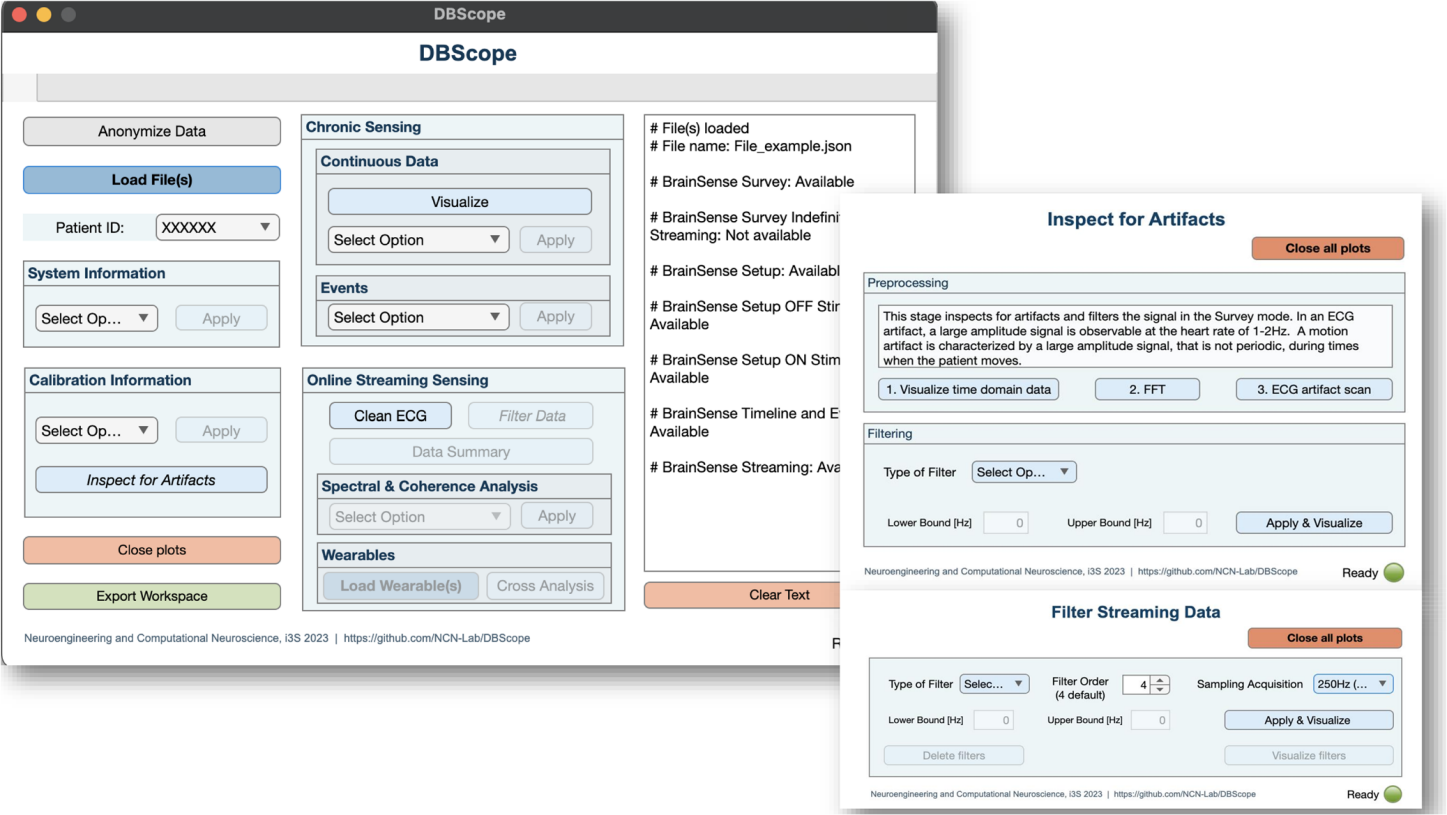
DBScope graphical interface. The toolbox comprises a main interface that accommodates four sections. Each section gives access to part of the data contained in the loaded files. Within the *Calibration Information* and *Online Streaming Sensing* sections, the user can access secondary interfaces, that are used in artifact inspection and signal filtering, respectively. The main interface also contains a text area that displays information during user interaction. For instance, upon loading a file, it shows the available sensing modes.

Integrating and analyzing information from multiple types of recordings can be troublesome and time-consuming. To facilitate this process, we developed an user interface (Fig. 1), which provides a graphical environment for data analysis and exploration. The development of this graphical user interface targets mainly an audience with no previous experience in programming, but who is keen on including DBS data analysis into their clinical or research routines. Noteworthy, DBScope also provides researchers and experienced programmers the possibility to create their own scripts in MATLAB, adapting or introducing new functions to better fit their scientific questions (Supplementary Fig. 1).

### Operational workflow

**Fig. 2:**
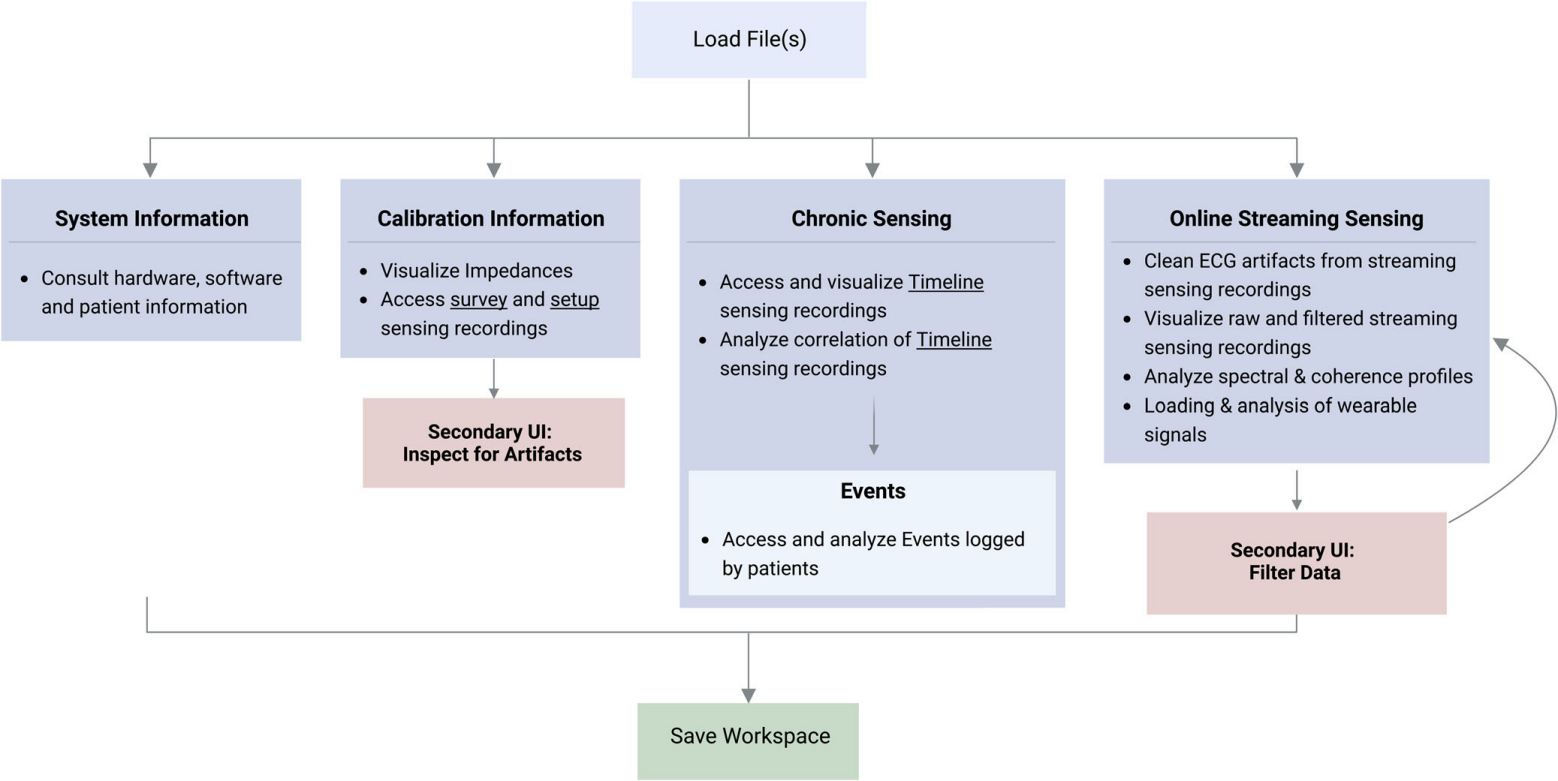
General workflow. The user starts by loading one or multiple files. The user can always return to this step, with the option to save the current workspace. After loading the file(s), four streams of operations are available: *System information*, gives information about the patient, the device, and the in-clinic visit; *Calibration Information* deals with the recordings obtained when testing and setting the stimulation and sensing parameters, and has a secondary interface for artifact inspection; Chronic Sensing offers multiple visual and analytical functions for the Timeline and Events recordings; Online Streaming Sensing provides tools for visualization, artifact removal, filtering (using a secondary interface), and alignment with wearable data. Regardless of the chosen *workflow*, the user can always save the workspace (for more information, consult the DBScope documentation).

The first step in DBScope is to load the files of interest. The toolbox is capable of handling one or multiple files from the same patient, facilitating combined/aggregated analysis as required. Once the files are loaded, four main streams of operations are suggested (Fig. 2), considering the sensing modes present in the loaded file(s):

- **System Information** provides information on the neurostimulator, the patient, and the in-clinic visit.
- **Calibration Information** gives access to the impedance tests, to the *Survey* and *Setup* recordings, and to a secondary interface for artifact exploration.
- **Chronic Sensing** offers visualization and analytical tools for the *Timeline* and *Events* recordings.
- **Online Streaming Sensing** draws on four main functionalities: 1) an ECG cleaning algorithm; 2) a filtering tool that calls a secondary interface; 3) spectral and correlation tools; and 4) a tool for visual cross-analysis with wearable data. For the latter, the user must load the corresponding wearable data files, in a CSV format (for more information, consult the DBScope documentation). During visualization, the toolbox automatically aligns the wearable recordings with the streaming recordings using the marked timestamps.

### Case-studies

To exemplify the application of the toolbox and showcase some of its features (see also Supplementary Fig. 2), we present two case studies.

**Fig. 3:**
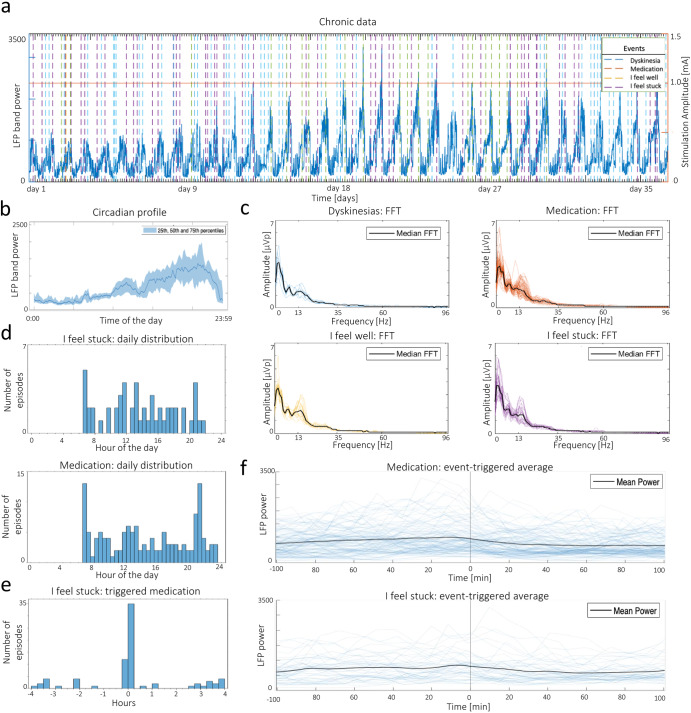
Case study of out-of-clinic data. **a** Representation of *Timeline* and *Events* of the selected hemisphere. The recordings cover approximately one month in a conventional DBS protocol (continuous fixed stimulation amplitude). **b** Estimated circadian profile of the *Timeline* recording in (**a**). The LFP power is higher during waking hours. **c** Fast Fourier Transform (FFT) profiles for each of the recorded *Events*. The observed median profiles were similar, with the highest variation around the 15 Hz peak. **d** Daily distribution of ‘Medication’ and ‘I feel stuck’ episodes. Notice that distributions are similar, with three periods of higher incidence (6 h, 11-12 h, 20-21 h). **e** Distribution of ‘I feel stuck’ events relative to ‘Medication’ events (triggered at zero). Interestingly, ‘I feel stuck’ episodes are mostly reported after ‘Medication’. **f** Event-triggered average plots showing the power of the selected band in the proximity of ‘Medication’ and ‘I feel stuck’ episodes. The mean power decreases after the two events.

The first case study addresses analysis of the long-term LFP dynamics (up to a month) of out-of-clinic recordings. Circadian and diurnal power fluctuations within the preselected frequency band can be observed throughout the entire 34-day *Timeline* recording (Fig. 3a, b). While beta power exhibits a consistent reduced magnitude overnight (which is indicative of sleep time), most fluctuations occur during daytime and increase in range over the recording period. The latter phenomenon may be explained by the fact that the implantation took place two weeks prior to the recording and the tissue around the implant was still stabilizing (i.e., inflammatory response, edema, and scar tissue formation persisted).

This patient’s *Events* recordings include ‘Medication’, ‘I feel well’, ‘I feel stuck’, and ‘Dyskinesias’ episodes (Fig. 3c. Dyskinetic episodes are characterized by lower theta-alpha and beta powers (Supplementary Fig. 3). The daily distribution of ‘I feel stuck’ episodes contains multiple periods of higher incidence, which are aligned with the medication intake distribution (Fig. 3d). Interestingly, most ‘I feel stuck’ episodes appear immediately after medication (Fig. 3e). While the inverse relationship would be expected (i.e., medication would decrease rigidity), it is possible that the patient was using the medication schedule as a cue to report the immediately preceding rigidity states.

Post-medication power shows a consistent decrease within a few minutes of medication intake (Fig. 3f). This phenomenon aligns with the known suppression of beta activity induced by Parkinsonian medication and supports the usefulness of this frequency band in investigating the wear-off effect. This decrease is also seen after ‘I feel stuck’ episodes, which is not surprising given the temporal relationship with medication episodes (Fig. 3e).

**Fig. 4:**
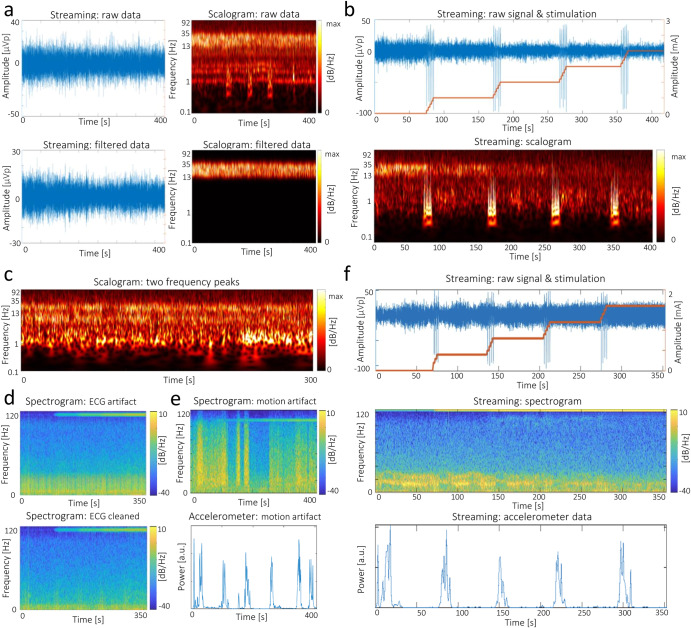
Case study of in-clinic recordings. **a** Application of a customized filter to better highlight a band of interest (13–35 Hz). **b** Raw neuronal recording and time-frequency analysis aligned to therapy changes. Amplitude stepping induces changes both at the raw signal (abrupt magnitude rises) and scalogram (high low-frequency activity). Note that the y-axis of the scalograms has a logarithmic scale. **c** Example of a recording where both beta and theta-alpha activities are elevated. **d** Recording with cardiac artifacts before (top) and after (bottom) cleaning. **e** Recording with motion-induced artifacts. The artifacts span across the entire frequency spectrum, being more intense at the peaks in motion power and vanishing once the motion power is plotted over zero. **f** Cross-signal analysis for a titration recording during a motor task. Both stimulation and movement suppress beta-band activity.

The second case study explores LFP dynamics in shorter time scales (in the domain of minutes) using the streaming (in-clinic) recordings of multiple patients. Scalograms, obtained with a wavelet transform method, exhibit bands with heightened activity for many patients (Fig. 4a, top plots). To enable easier analysis and interpretation aligned with clinical observations, a filtering tool can be applied to highlight and isolate these bands (Fig. 4a, bottom plots). Instead of merely displaying the overall power of the selected band (like the ‘programming’ tablet), it exhibits distinct frequency variations within the band. Moreover, it provides greater flexibility by allowing the highlighting of multiple bands, which is advantageous in situations where monitoring a single band may not be sufficient for therapy evaluation (e.g., when the theta-alpha bands are also elevated, Fig. 4c).

In many of the recordings, the stepping of stimulation amplitude resulted in abrupt rises of the LFP signal, evident by an increase in lower frequency oscillations (Fig. 4b). This phenomenon has an artifactual nature and has been previously reported in Percept PC patients^18^. Incidentally, artifacts in DBS are common and can arise from various sources, including cardiac activity (Fig. 4d, top plot), motion (Fig. 4e), and stimulation itself. Furthermore, it is not uncommon for a signal to be contaminated by multiple types of artifacts. In some recordings, the ECG cleaning tool can successfully remove the ECG artifact (Fig. 4d, bottom plot).

In some patients, an increase in stimulation amplitude results in beta-band activity suppression both at rest (additional case study, Supplementary Fig. 4) and during motor performance (Fig. 4f). Additionally, desynchronization of beta activity is shown to occur at lower stimulation amplitudes during a motor task involving the opening and closing of the contralateral fist.

## Discussion

In this work, we address the need of accessible and comprehensive analytical tools for the DBS therapies. We present a toolbox with an intuitive user interface that allows both clinicians and researchers to access and explore intricate electrophysiological data. To the best of our knowledge, no other toolbox offers a comparable range of functions in terms of both extent and variety, together with ease to run/setup and customize (but see ref. ^17^). Among these functions, we would like to emphasize the inclusion of an ECG cleaning tool and the capability to visualize streaming recordings with data obtained from wearable devices. Furthermore, the toolbox operates without the need for an internet connection and patient data is not required to be stored on a server. While we acknowledge that a central database may be desirable in a healthcare setting, it is also true that it imposes additional technical/security measures regarding the sharing of personal data.

To standardize some of the most performed analyses of the field, we designed a novel structure of classes and developed a diverse repertoire of functions within each class. Some of these functions were adapted from existing toolboxes, notably in the detection and cleaning of cardiac artifacts. By adopting an object-oriented programming framework, we were able to produce a toolbox that can analyze multiple files in a simple and intuitive workflow. This is desirable not only from a software design perspective, but also from a scientific perspective, where longitudinal, multi-signal and multi-patient analyses are relevant^11,19,20^.

To illustrate the application of DBScope, we present two case studies that concentrate on out-of-clinic and in-clinic recordings. The latter are more commonly used in the context of Percept PC, as they offer a higher temporal resolution. Nevertheless, out-of-clinic recordings still provide an invaluable glimpse into real-world settings, where data-driven DBS therapies aim to actuate. In fact, we observed that chronic data contains valuable information, such as the patients’ circadian patterns, which exhibit longer temporal dynamics. These patterns are challenging to identify in in-clinic recordings but are discernible in long-term ones. At the same time, it was possible to study the response of the LFP to specific occurrences, such as medication intake or rigidity episodes. While the marking of medication episodes showed potential in monitoring the wear-off effect, other events were reported less times and typically coincided with medication intake, suggesting that the patient was reporting retroactively. Regarding the in-clinic recordings, the toolbox offered clear visualizations that facilitated the identification of both artifacts and clinically relevant frequency bands. Additionally, the possibility of aligning streaming recordings with wearable data proved to be a relevant addition to DBScope, enabling the investigation of movement-related modulations.

Although DBScope succeeded on many fronts, it bears some limitations. Presently, the toolbox operates exclusively on files extracted from Medtronic’s Percept PC setup. As new devices with sensing capabilities have since emerged (Medtronic’s Summit RC + S and Newronika’s AlphaDBS), we are considering the development of specific parsers to accommodate them. DBScope is also reliant on commercial software (MATLAB). However, it should be noted that universities and research institutes often offer campus-wide licenses to their members.

Future developments aim to address two challenges. The first lies in the search for biomarkers. Defining a biomarker, or library of biomarkers, is an essential, albeit complex, step for the development of patient- and symptom-specific DBS therapies^21–23^. A direct mapping between brain activity and reported symptoms is seldom possible, due to the subjective and elusive natures of the latter. In these cases, a second signal, more interpretable and highly linked with the symptom type, is often introduced to facilitate the mapping process. For instance, accelerometry data is widely used to link electrophysiological signals with motor symptoms. In light of this, we have already included the functionality to load accelerometry data into DBScope and are actively developing additional methods to leverage the information contained in these signals. The second challenge stems from the fact that LFP are prone to artifacts of different origins, such as cardiac, movement, and stimulation. Although current devices have implemented artifact-cleaning algorithms, not all artifacts can be reliably identified and effectively cleaned. DBScope currently enables artifact screening in an iterative process, where the user alternates between visualization and filtering/cleaning steps. However, this approach is time-consuming and ineffective when the entire frequency spectrum is affected. In this respect, we are invested in the development of algorithms that not only automatically detect the source of these artifacts, but also clean them accordingly.

One of our major goals was to create the conditions for the DBS community to adopt and easily contribute to the enhancement of this toolbox, in response to advancements and discoveries in DBS research. For this reason, DBScope is “open source” and is accessible through an online repository. The dependence on MATLAB was circumvented with the creation of standalone applications for both Microsoft Windows and macOS operative systems. These standalone applications, also available in the online repository (“Release” section), allow DBScope to be used without having to install MATLAB (royalty-free). We firmly believe that these types of initiatives are key in fostering the emergence of novel methods for clinical integration. Although we are currently working on updates, we encourage the clinical and research communities to adapt the tools and algorithms already available in DBScope and to share their insights, becoming part of this joint effort to improve the DBS therapy.

In conclusion, DBScope is an open-source computational toolbox to import, visualize and analyze files from the Percept PC device (Medtronic, BrainSense Technology). The toolbox can be used programmatically or through an interface for users without programming experience. This way, it can be directly integrated into the clinical and research practices, whilst remaining adaptable to new research questions. Its functionalities are up to date with current literature standards for the evaluation of LFP in DBS. Moreover, future updates are in store. Overall, DBScope is a versatile tool focused on the widespread improvement of data-driven DBS therapies, by expanding the accessibility of the data and by promoting new forms of analyzing the complexity of DBS data. DBScope is available for download in GitHub, and can be accessed via this link: https://github.com/NCN-Lab/DBScope.

## Methods

### Sensing data

The initial motivation for the DBScope toolbox stemmed from the interest in conducting in-depth analysis on the files extracted from the Medtronic’s Percept PC setup. Besides information regarding the in-clinic visit, the patient and the neurostimulator, these files contain two main groups of recordings: the in-clinic recordings, which are subdivided in *Survey*, *Setup*, and *Streaming* sensing modes; and the chronic (or out-of-clinic) recordings, containing the *Timeline* and the *Events* sensing modes (up to 60 days for the Percept PC device).

### Ethics approval and consent information

Fully anonymized recordings from patients already implanted with the Percept PC neurostimulator with sensing capabilities were used to develop, test and validate the DBScope toolbox. We have obtained informed consent from the patients and the study received authorization (reference CE 233/2023) from the Ethics Committee of Centro Hospitalar Universitário de São João (São João University Hospital Center), Portugal. As such, this clinical dataset is not shared. However, a fully anonymized recording is provided with the DBScope toolbox for testing purposes.

### Toolbox features

**Fig. 5:**
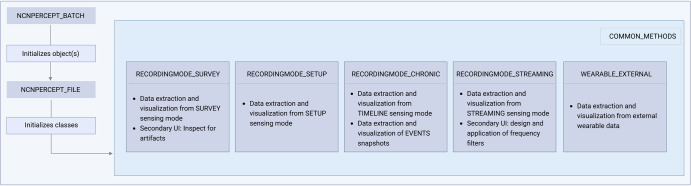
DBScope internal structure*.* The toolbox was designed using an object-oriented programming framework. Upon initialization, the class NCNPERCEPT_BATCH separates the loaded files in patients (each containing one or several files). Each file gives rise to a NCNPERCEPT_FILE object. These objects contain six classes, each with its own properties and methods. Each class is named after the type of recording that it can access. The WEARABLES_EXTERNAL class handles additional data that is not present within the neurostimulator files and needs to be loaded separately afterward. COMMON_METHODS is an auxiliary class that runs background methods.

DBScope was developed with MATLAB 2022b (MathWorks, Natick, MA), a well-established scientific programming language with several built-in tools, ranging from signal analysis to user interface creation. DBScope was designed with a set of core features in mind: 1) interactivity, as to ensure that users with little to no programming experience could use it in their practices; 2) reusability, so that users with some programming experience could easily create or adapt the tools to better fit their research questions; 3) integration, to allow the simultaneous analysis of both different signals (in cases where motor data is also acquired) and multiple files (for longitudinal studies); 4) customization, to the extent that each sensing mode should have tailored methods. We used an object-oriented programming paradigm to facilitate the fulfillment of these requirements (Fig. 5). Specific functions in DBScope were derived from pre-existing open-source computational toolboxes: the extraction of LFP and stimulation information methods are based on the analogous functions present in the Thenaisie et al. toolbox^13^; the electrocardiographic (ECG) artefact cleaning tool was extracted from the Perceive toolbox^14^. Regarding exportation, the toolbox offers two options: 1) a MATLAB file organized in the DBScope internal structure; and 2) a set of MATLAB files that are compatible with FieldTrip, each corresponding to one type of sensing mode.

### Supplementary information

Supplementary information

DBScope demonstration video

## Data Availability

No datasets were specifically generated for this study. A fully anonymized Percept PC (Medtronic) recording is provided with the DBScope toolbox for testing purposes.

## Code availability

The underlying code for this article, together with a fully anonymized recording file for testing/demonstration purposes, is available in GitHub and can be accessed via this link: https://github.com/NCN-Lab/DBScope.
