# Supplementary material for: DBScope as a versatile computational toolbox for the visualization and analysis of sensing data from deep brain stimulation
Source: NPJ Parkinsons Dis. 2024 Jul 15;10:132. doi: 10.1038/s41531-024-00740-z (PMC11251161; doi:10.1038/s41531-024-00740-z)
Supplement: Supplementary file 1 — Supplementary information [file 41531_2024_740_MOESM1_ESM.pdf]

SUPPLEMENTARY INFORMATION

DBScope as a versatile computational toolbox for the visualization and analysis of sensing data from Deep Brain Stimulation

Andreia M. Oliveira[1,2,†], Eduardo Carvalho[1,3,†], Beatriz Barros[1,2], Carolina Soares[4,5], Manuel Ferreira-Pinto[4,5], Rui Vaz[4,5], Paulo Aguiar[1,2,4,*]

[1] Neuroengineering and Computational Neuroscience Lab, Instituto de Investigação e Inovação em Saúde (i3S) - University of Porto, Portugal

[2] Faculty of Engineering of University of Porto (FEUP), Portugal

[3] ICBAS – School of Medicine and Biomedical Sciences – University of Porto, Portugal

[4] Faculty of Medicine of University of Porto (FMUP), Portugal

[5] Centro Hospitalar Universitário de São João (CHUSJ), Portugal

[†] Equal contribution

[*] Corresponding author: pauloaguiar@i3s.up.pt

```matlab
%% DBScope example script
% Initialize data object
data = NCNPERCEPT_BATCH;
[ status, text ] = data.open_batch_files();
% Exemplify with one file
patient = 1; file = 1;
% Disp Lead Information
info = data.ncnpercept_patient{patient}{file}.getLeadConfig();
% Chronic - Timeline
data.ncnpercept_patient{patient}{file}.chronic_obj.plotLFPTrendLogs();
% Chronic - Circadian Distribution
data.ncnpercept_patient{patient}{file}.chronic_obj.plotCircadian();
% Online Streaming - ECG Artifact Cleaning
data.ncnpercept_patient{patient}{file}.streaming_obj.cleanECG(250);
% Online Streaming - Data Summary
data.ncnpercept_patient{patient}{file}.streaming_obj.plotSummaryStreaming(1);
```

Supplementary Figure 1. DBScope *script example.* The initial steps involve initializing the mother object, NCNPERCEPT_BATCH, followed by the file(s) loading. Subsequently, one file is analyzed considering three different streams of operations: Calibration System, for the display of the lead information; Chronic Sensing, for the visualization of the Timeline and circadian distribution; and Online Streaming Sensing, for the application of the ECG artifact cleaning algorithm and visualization of the data summary.

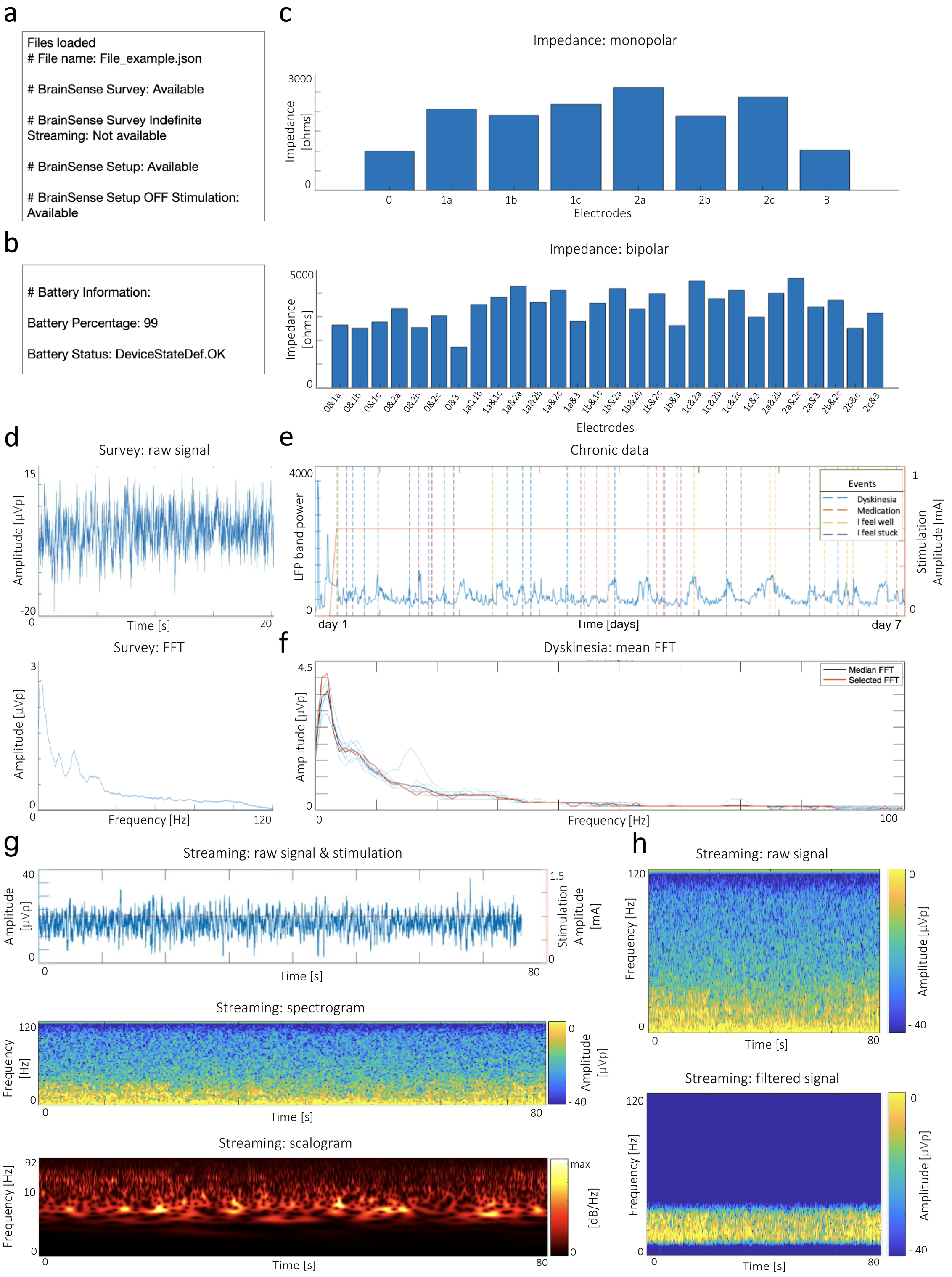

Supplementary Figure 2: Overview of the toolbox outcomes. (a) Snapshot of the text window displayed once a file is loaded. **(b)** Snapshot of the battery information. **(c)** Impedance information (monopolar and bipolar) for one hemisphere, accessible in the Calibration section. **(d)** Raw signal and Fast Fourier Transform (FFT) of a Survey recording. **(e)** Chronic data of one hemisphere, with the events marked by the patient. **(f)** FFT of 'Medication episodes marked in **(e)**. **(g)** Data summary, containing the raw signal,

the stimulation amplitude, the spectrogram, and the scalogram obtained with a wavelet method of an Online Streaming recording. **(h)** Spectrograms of the recording of **(g)** before and after a bandpass filter.

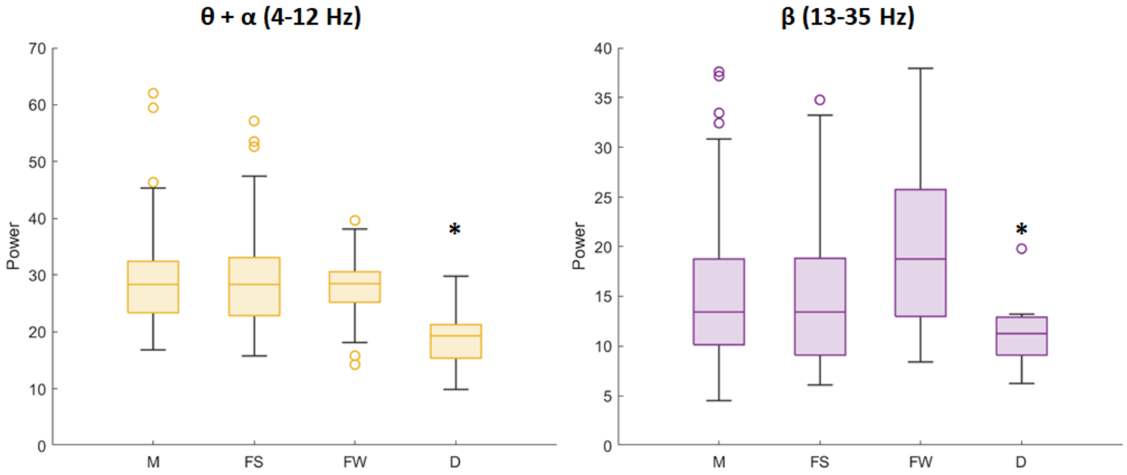

Supplementary Figure 3. Power in alpha-theta and beta frequency bands of four different events. Each graph has the minimum, 25th percentile, median, 75th, and maximum values for the four types of events: M – Medication; FS – Feeling stuck; FW – Feeling well; D – Dyskinesias. The asterisk marks the instances where dyskinesias are statistically different from at least one other event (ANOVA, p<0.05).

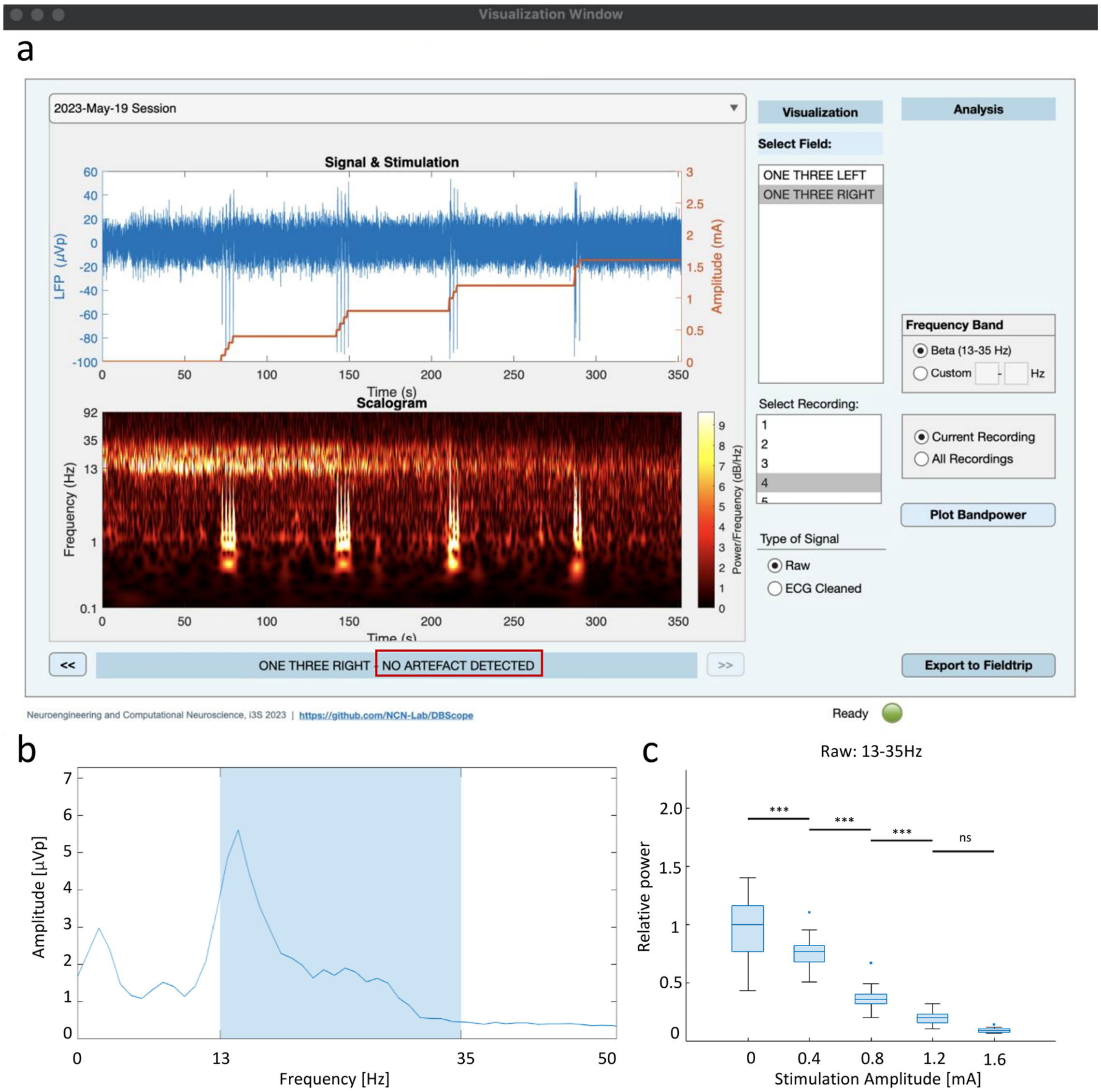

Supplementary Figure 4. Application of DBScope in a clinical evaluation. (a) Snapshot of the DBScope Morlet Wavelet function. In the absence of detected artifacts (highlighted in a red box), the raw data was selected. The plot displays a screenshot of the toolbox output, featuring the following: top row – raw LFP traces and stimulation; and bottom row – scalogram of the signal. Notably, the reduction of the beta band on the third level of stimulation is observable from the third to fifth stimulation levels, suggesting a response to the stimulation. **(b)** FFT of the selected sensing channel, 'ONE THREE RIGHT', with a clear peak in the low beta band. **(c)** Computation of the relative bandpower for the beta band (13-35Hz) reveals statistical significance (One-way ANOVA, $p<0.001$) among most stimulation levels. The statistical differences between stimulation levels are shown with the horizontal bars; there is no significant (ns) difference between levels 1.2 and 1.6 nA.

Supplementary Movie 1. Screen-capture video with demonstration of DBScope use. This video highlights important functions/features of DBScope, from opening a recording json file, inspecting different system and calibration data, up to visualization of chronic and streaming data.
